# Supplementary material for: RITAN: rapid integration of term annotation and network resources
Source: PeerJ. 2019 Jul 19;7:e6994. doi: 10.7717/peerj.6994 (PMC6644632; doi:10.7717/peerj.6994)
Supplement: Supplemental Information 1 — This archive contains five vignettes showing aspects of RITAN’s use. They are included for completeness of this manuscript as a stand-alone work. For the very latest versions, see the project’s GitHub page and/or the devel branch of bioconductor.org. [file peerj-07-6994-s001.zip › choosing_resources.html]

Choosing Resources


# Choosing Resources

#### *Michael T. Zimmermann*

#### *2018-10-10*

RITAN indexes multiple resources and choosing which of them are most relevant for your study can be a challenge. To help with this process, we provide below a set of examples from different types of studies and the thinking behind which resources were used.

# Resources Indexed by default

```
library(RITANdata)
library(RITAN)
library(knitr)
kable( attr(network_list, 'network_data_sources') )
kable( sapply(geneset_list, length), col.names = c('# Genesets') )
```

While many resources contain information about protein complexes (obligate interactions) and protein-protein interactions (often transient), some use experimental techniques that are specific for physical ineractions. Determining whihc resources indicate physical, through-metabolite, and through-DNA (i.e. transcription factors) interactions, we recommend: 1) each resource’s primary publication 2) the pathguide website 3) the following guidelines

# Example: Protein Complexes & Protein-Protein Interaction (PPI)

```
genes <- geneset_list$MSigDB_C5$APICAL_JUNCTION_COMPLEX
e <- network_overlap( genes, resources = c('CCSB','STRING'), minStringScore = 700 )
## We also strongly encourage use of the BioPlex database, which we do not distribute with RITAN in compliance with their licensing.
```

# Example: Metabolic Network Interaction

```
genes2 <- geneset_list$MSigDB_C5$AMINE_METABOLIC_PROCESS
e2 <- network_overlap( genes, resources = c('PID','HumanNet') )
```

# Example: Expression Signalling

```
genes2 <- geneset_list$MSigDB_C5$AMINE_METABOLIC_PROCESS
e2 <- network_overlap( genes, resources = c('ChEA','HumanNet') )
```
