# Supplementary material for: RITAN: rapid integration of term annotation and network resources
Source: PeerJ. 2019 Jul 19;7:e6994. doi: 10.7717/peerj.6994 (PMC6644632; doi:10.7717/peerj.6994)
Supplement: Supplemental Information 1 — This archive contains five vignettes showing aspects of RITAN’s use. They are included for completeness of this manuscript as a stand-alone work. For the very latest versions, see the project’s GitHub page and/or the devel branch of bioconductor.org. [file peerj-07-6994-s001.zip › enrichment.html]

Enrichment Vignette


# Enrichment Vignette

#### *Michael T. Zimmermann*

#### *2018-10-10*

# Introduction to RITAN

RITAN is an R package for Rapid Integration of Term Annotation and Network resources. It is to be applied to a list of gene symbols - for example, those identified by differential gene expression analysis.

The prevalence of gene expression analysis has led to the common outcome: Researchers identified a list of genes-of-interest and are wondering how they fit together. A common example would be a research group studying the effect of a treatment and identifying the differentially expressed genes between the treatment and control groups. Two logical questions are, “What functions are achieved by this group of genes?” and “How do these genes work together to achieve that function?” The RITAN package consolidates multiple annotation resources to help answer both questions.

# Quick Start

Our process of rapidly leveraging a comprehensive set of functional annotations begins from a list of prioritized genes. RITAN is distributed with example genesets indexed by MSigDB. For this example we chose a study of influenza response under multiple conditions. In this vignette we will demonstrate analysis across multiple annotation resources. This can be achieved in one function call as exemplified in the following “quick start” example of RITAN’s annotation capabilities.

```
library(RITANdata)
library(RITAN)
```

Users may use RITAN to query multiple annotation resources simultaneously, and performing FDR-adjustment across the combined set:

```
# We define a query using gene symbols
my_genes <- c('ABCA2','ACAT2','ACSS2','CD9','CPEB2','CTNNB1','FASN','LDLR','LPL','LSS')

# Now, you can rapidly get enrichment results by:
# e <- term_enrichment(my_genes)

# However, we will make two simplifications here, for expediency.
#  1) only look at two resources (of the many available)
#  2) use a cached background gene list of human protien coding genes
resources <- c("ReactomePathways", "MSigDB_Hallmarks")
e <- term_enrichment(my_genes, resources = resources, all_symbols = cached_coding_genes)
```

```
## Loading the requested genesets of "ReactomePathways"...
##  Loading the requested genesets of "MSigDB_Hallmarks"...
## 
##  Loaded 1755 genesets.
##                                                                 name
## 1708               MSigDB_Hallmarks.HALLMARK_CHOLESTEROL_HOMEOSTASIS
## 268            ReactomePathways.Chylomicron-mediated lipid transport
## 797                          ReactomePathways.Lipoprotein metabolism
## 837           ReactomePathways.Metabolism of lipids and lipoproteins
## 85   ReactomePathways.Activation of gene expression by SREBF (SREBP)
##                 p  n n.set            q
## 1708 3.611330e-25 10    73 6.337884e-22
## 268  3.362719e-05  2    17 2.950786e-02
## 797  1.071665e-04  2    30 6.183554e-02
## 837  1.950507e-04  4   616 6.183554e-02
## 85   2.013932e-04  2    41 6.183554e-02
```

```
summary(e)
```

```
##                                                                 name
## 1708               MSigDB_Hallmarks.HALLMARK_CHOLESTEROL_HOMEOSTASIS
## 268            ReactomePathways.Chylomicron-mediated lipid transport
## 797                          ReactomePathways.Lipoprotein metabolism
## 837           ReactomePathways.Metabolism of lipids and lipoproteins
## 85   ReactomePathways.Activation of gene expression by SREBF (SREBP)
## 1254              ReactomePathways.Retinoid metabolism and transport
##                 p  n n.set            q
## 1708 3.611330e-25 10    73 6.337884e-22
## 268  3.362719e-05  2    17 2.950786e-02
## 797  1.071665e-04  2    30 6.183554e-02
## 837  1.950507e-04  4   616 6.183554e-02
## 85   2.013932e-04  2    41 6.183554e-02
## 1254 2.114036e-04  2    42 6.183554e-02
```

*Please cite each resource used in your analysis and register at MSigDB if you use their data. Doing so will help to ensure the future availability and extension of these valuable resources.*

# Full Example of Enrichment Analysis in RITAN

This version of RITAN is distributed with the following annotation resources:

```
names(geneset_list)
```

```
##  [1] "MSigDB_Hallmarks"                 "MSigDB_C2"                       
##  [3] "MSigDB_C3.TFtargets"              "MSigDB_C3.miRNA"                 
##  [5] "MSigDB_C5"                        "MSigDB_C7"                       
##  [7] "GO"                               "GO_slim_PIR"                     
##  [9] "GO_slim_generic"                  "PathwayCommonsPathways"          
## [11] "ReactomePathways"                 "NetPath_Gene_regulation"         
## [13] "Chaussabel_Modules"               "Blood_Translaiton_Modules"       
## [15] "KEGG_filtered_canonical_pathways" "DisGeNet"
```

Each resource is a list of gene symbols. For example:

```
head(geneset_list$MSigDB_C7$GSE9988_LPS_VS_LOW_LPS_MONOCYTE_UP)
```

```
## [1] "A2M"       "ABCA9"     "ADAM20"    "AMPH"      "ANKRD26P3" "ANKRD29"
```

We now demonstrate the typical use-case of term enrichment: Annotating a list of genes-of-interest by the biologic functions or pathways they participate in. Your genes-of-interest could come from many types of analysis, such as differential gene expression.

The “geneset\_list” object included in the package, contains multiple annotation resources defined by the gene symbols from each resource. We select an example from a study of “Innate Immune responses to TREM-1 Activation” deposited in GEO under GSE9988 and with the top deferentially expressed genes under each condition indexed by MSigDB. These MSigDB genesets are included within RITAN under the “C7” modules.

```
selection <- grepl( 'GSE9988_(LOW_)*LPS_VS_.+UP', names(geneset_list$MSigDB_C7), perl=TRUE )
study_set <- geneset_list$MSigDB_C7[selection]
str(study_set)
```

```
## List of 7
##  $ GSE9988_LPS_VS_LOW_LPS_MONOCYTE_UP               : chr [1:199] "A2M" "ABCA9" "ADAM20" "AMPH" ...
##  $ GSE9988_LPS_VS_LPS_AND_ANTI_TREM1_MONOCYTE_UP    : chr [1:200] "ACOX1" "ACSL1" "ACVR2A" "ADA" ...
##  $ GSE9988_LPS_VS_CTRL_TREATED_MONOCYTE_UP          : chr [1:200] "ACSL1" "ADAMDEC1" "ADM" "ANO5" ...
##  $ GSE9988_LPS_VS_VEHICLE_TREATED_MONOCYTE_UP       : chr [1:200] "ABL2" "ACSL1" "ADM" "ADRB2" ...
##  $ GSE9988_LOW_LPS_VS_ANTI_TREM1_AND_LPS_MONOCYTE_UP: chr [1:200] "ACSL1" "ACVR2A" "ADA" "ADH1B" ...
##  $ GSE9988_LOW_LPS_VS_CTRL_TREATED_MONOCYTE_UP      : chr [1:200] "ACSL1" "ADAMDEC1" "ADM" "ANO5" ...
##  $ GSE9988_LOW_LPS_VS_VEHICLE_TREATED_MONOCYTE_UP   : chr [1:200] "ABL2" "ACSL1" "ADM" "ADRB2" ...
```

From the above, we can see that 7 lists of gene symbols have been selected and that each is the result of comparing one treatment group to another and identifying the 200 most differentially expressed genes. The treatments used that we will consider in this example are LPS, Anti-TREM1 Antibody, Vehicle-only, and IgG Control.

The default set of annotation resources consists of GO, Reactome Pathways, KEGG Filtered Canonical Pathways, and MSigDB Hallmarks. Thus, by default, a total of 17,724 functional terms and pathways will be considered by RITAN.

The function term\_enrichment\_by\_subset() uses these annotation resources to evaluate enrichment simultaneously across each set of input gene lists. In this example those gene lists contain the genes differentially expressed between each condition. Note that we have loaded all 4 resources together, making the q-value adjustment across them. For brevity, we will consider a filtered set of Canonical Pathways and Hallmark functions.

```
e <- term_enrichment_by_subset( study_set, q_value_threshold = 1e-5, 
                                resources = resources,
                                all_symbols = cached_coding_genes )
```

```
## Loading the requested genesets of "ReactomePathways"...
##  Loading the requested genesets of "MSigDB_Hallmarks"...
## 
##  Loaded 1755 genesets.
##  Running Enrichment...FDR adjustment...done.
```

You can see that between these two resources, 188 pathways were tested for enrichment by RITAN. We identify the representation (or enrichment) of each, across the input study sets. After performing false discovery adjustment across the group, we save the significant associations (in this case, q < 10-5).

We next plot a heatmap of the enrichment results:

```
plot( e, show_values = FALSE, label_size_y = 7, label_size_x = 7 )
```

The plot shows four of the study results with highly significant enrichment for “Hallmark of TNF-alpha signaling via NFKB.” The enrichment is so strong that it dominates the shading of the heatmap as the shading scales by statistical significance; values shown are -log10(q-value).

In order to make better use of the color scale, we set the “cap” parameter to 10 such that the q-values more significant than 1x10^-10^ are “capped” at a value of 1x10^-10^. This results in a more informative visualization.

```
plot( e, show_values = FALSE, label_size_y = 7, label_size_x = 7, cap=10 )
```

Importantly, the full data is available to the user. For simplicity, we print the data for the top 3 terms, but omit the column names as they are long and visible in the above plot.

```
prmatrix( e[1:3,], collab = c('name','n.set',1:7) )
```

```
##      name                                                n.set 1   2     
## 1706 "MSigDB_Hallmarks.HALLMARK_TNFA_SIGNALING_VIA_NFKB" "198" "0" "18.1"
## 1736 "MSigDB_Hallmarks.HALLMARK_INFLAMMATORY_RESPONSE"   "197" "0" " 8.2"
## 1715 "MSigDB_Hallmarks.HALLMARK_APOPTOSIS"               "159" "0" " 0.8"
##      3      4      5      6      7     
## 1706 "81.8" "83.6" "17.4" "92.8" "87.3"
## 1736 "19.7" "20.9" "17.4" "22.0" "22.2"
## 1715 " 8.2" " 8.2" " 1.8" "10.4" " 7.2"
```

## Add an Annotation Matrix

An annotation matrix for which gene list can facilitate a visual evaluation of relationships between sample or study conditions and enrichment. In this instance, we could parse the name of each geneset for information about it, but for simplicity, we manually construct the matrix.

```
mat <- matrix(c("LPS","LPS","LPS","LPS","LOW_LPS","LOW_LPS","LOW_LPS",
                "LOW_LPS","LPS_AND_ANTI_TREM1","CTRL_TREATED",
                "VEHICLE_TREATED","ANTI_TREM1_AND_LPS","CTRL_TREATED","VEHICLE_TREATED"),
                nrow = 2, byrow = TRUE )
rownames(mat) <- c('Condition1','Condition2')
colnames(mat) <- sprintf('Sample%s', 1:7)
print(mat)
```

```
##            Sample1   Sample2              Sample3        Sample4          
## Condition1 "LPS"     "LPS"                "LPS"          "LPS"            
## Condition2 "LOW_LPS" "LPS_AND_ANTI_TREM1" "CTRL_TREATED" "VEHICLE_TREATED"
##            Sample5              Sample6        Sample7          
## Condition1 "LOW_LPS"            "LOW_LPS"      "LOW_LPS"        
## Condition2 "ANTI_TREM1_AND_LPS" "CTRL_TREATED" "VEHICLE_TREATED"
```

```
plot( e, show_values = TRUE, label_size_y = 7, label_size_x = 7, cap=10, 
      annotation_matrix = mat, grid_line_color = 'black' )
```

### Show the Number of Genes

While enrichment accounts for the size of each geneset and the number of genes selected, in generating a significance score, sometimes it is informative to know also the counts.

```
n <- term_enrichment_by_subset( study_set, q_value_threshold = 1e-5,
                                resources = resources,
                                all_symbols = cached_coding_genes,
                                display_type = 'n', phred = FALSE )
```

```
## Loading the requested genesets of "ReactomePathways"...
##  Loading the requested genesets of "MSigDB_Hallmarks"...
## 
##  Loaded 1755 genesets.
##  Running Enrichment...FDR adjustment...done.
```

```
plot( n, show_values = TRUE, label_size_y = 7, label_size_x = 7, cap = 20 )
```

Thus, we generate a similar plot as previous, but now the number of genes overlapping each term or pathway is displayed.

# Term Enrichment

This function uses all terms within a set of resources, the default consisting of GO, ReactomePathways, KEGG\_filtered\_canonical\_pathways, MSigDB\_Hallmarks, to evaluate the level of overlap with an input vector of gene names. The output of the term\_enrichment function is a data frame that contains information about how well the input gene symbols overlap with known annotation data sets. The first field in the data frame is the name of the known gene set. The second field is the hypergeomtric p value computed from the number of gene names in the test gene set that are also in the published gene set. The third field contains the number of genes names that overlap both the test gene set and the published gene set. The fourth field contains the number of genes in the published gene set. The fifth field gives the list of gene names in common between the two gene sets and the sixth field is a measure of the false discovery rate derived from the p value.

Input resources are either taken from our pre-loaded list of resources or an input GMT file. For an explanation of the GMT file format, see the GSEA help page

```
data("vac1.day0vs31.de.genes")
te <- term_enrichment( geneset = vac1.day0vs31.de.genes )
```

The R object, geneset\_list, included with the package contains 15 possible annotation annotation resources. They are: MSigDB\_Hallmarks, MSigDB\_C2, MSigDB\_C3.TFtargets, MSigDB\_C3.miRNA, MSigDB\_C5, MSigDB\_C7, GO, GO\_slim\_PIR, GO\_slim\_generic, PathwayCommonsPathways, ReactomePathways, NetPath\_Gene\_regulation, Chaussabel\_Modules, Blood\_Translaiton\_Modules, KEGG\_filtered\_canonical\_pathways, DisGeNet .

The term\_enrichment function can be modified to produce results from different gene sets by setting the “resources” parameter to one or more of these annotation resources.

# Term Enrichment Using Other Resources

The term\_enrichment function can be modified to produce results from different gene sets by setting the “resources” parameter to one or more of the annotation resources available to RITAN.

```
e <- term_enrichment( geneset = vac1.day0vs31.de.genes, verbose = TRUE, 
                      resources = c("Blood_Translation_Modules", "MSigDB_C7") )
```

An efficient way to include all available resources would be:

```
e <- term_enrichment( geneset = vac1.day0vs31.de.genes, verbose = TRUE, 
                      resources = names(geneset_list) )
```

\*However, one must be cautious about the appropriateness of doing so. Running enrichment across all available sources will provide a comprehensive view of what biologic functions your list of genes may influence, but it is likely that the terms across resources are related to one another. For instance, there are GO terms that closely correspond to certain pathway functions. The non-independence of terms could make false discovery adjustment too conservative. Point being: The broad stroke can be helpful, but the more focused analysis will likely be clearer and more effective.

# Term Enrichment for User-Input Resource

Below, we will load a user-defined resource generated from DisGeNet. DisGeNet is a resource of disease-gene relationships. We have arranged the DisGeNet data such that each disease name is treated as a “term.” This way, we can query if our genes-of-interest are enriched for association with disease states.

As an example, we will use genes-of-interest from a previous study of the differential expression of influenza infection which has been deposited in MSigDB.

```
gs  <- geneset_list$MSigDB_C7[['GSE6269_HEALTHY_VS_FLU_INF_PBMC_UP']]
gmt <- system.file("extdata", "curated_gene_disease_associations.gmt.gz", package="RITAN")

# -->> Not running here for brevity
# geneset_list$DisGeNet <- readGMT(gmt)
# str(head(geneset_list$DisGeNet))
```

The format of the gmt file is a simple tab-delimited style where the first column is the term name, the second is a reference to its origin, and all columns following are the genes associated with that term.

| Disease | Source | Gene1 | Gene2 | Gene3… |
| --- | --- | --- | --- | --- |
| Dermatitis, Allergic Contact | umls:C0162820 | NAMPT | CALCRL | IGSF6 |
| Hyperlipoproteinemia Type II | umls:C0020445 | CETP | ABCA1 | EPHX2 |
| Disease Progression | umls:C0242656 | CDH2 | ABCF2 | TRAP1 |
| Lymphoma, Non-Hodgkin | umls:C0024305 | CSF | 2 CSF3 | CYP2E1 |

Now, run the term enrichment for disease association.

```
e2  <- term_enrichment( gs, resources = gmt, all_symbols = cached_coding_genes )
```

```
## Loading C:/Users/mtzimmermann/AppData/Local/Temp/RtmpEv9QHt/Rinst2988151553d3/RITAN/extdata/curated_gene_disease_associations.gmt.gz
## 
##  Loaded 604 genesets.
##                                                 name           p n n.set q
## 471 Precursor T-Cell Lymphoblastic Leukemia-Lymphoma 0.008265793 2    17 1
## 144                                   Celiac Disease 0.009249671 2    18 1
## 465                                   Drug Eruptions 0.009249671 2    18 1
## 420                                 Hypersensitivity 0.011232428 3    57 1
## 459                                         Cataract 0.011365054 2    20 1
```

```
print( e2[1:3,-5] )
```

```
##                                                 name           p n n.set q
## 471 Precursor T-Cell Lymphoblastic Leukemia-Lymphoma 0.008265793 2    17 1
## 144                                   Celiac Disease 0.009249671 2    18 1
## 465                                   Drug Eruptions 0.009249671 2    18 1
```

From the default output which includes the top 5 most enriched terms, you can see that few terms are significantly enriched.

In order to better understand the resource and if it is a comprehensive definition of influenza infection, we can read in the GMT file and show the genes that define “Influenza” within DisGeNet:

```
geneset_list$DisGeNet <- readGMT(gmt)
print( geneset_list$DisGeNet[['Influenza, Human']] )
```

```
##  [1] "APOL6"    "ATF3"     "CCL2"     "CCL8"     "CXCL10"   "DDX58"   
##  [7] "DDX60"    "EIF2AK2"  "GBP1"     "HERC5"    "HERC6"    "IFI27"   
## [13] "IFI35"    "IFI44"    "IFI44L"   "IFI6"     "IFIH1"    "IFIT1"   
## [19] "IFIT2"    "IFIT3"    "IFIT5"    "IFITM3"   "IRF7"     "ISG15"   
## [25] "LAMP3"    "LY6E"     "MX1"      "MX2"      "OAS1"     "OAS2"    
## [31] "OAS3"     "OASL"     "PARP12"   "PLSCR1"   "RSAD2"    "RTP4"    
## [37] "SAMD9"    "SCO2"     "SERPING1" "SIGLEC1"  "SLC22A8"  "STAT1"   
## [43] "TDRD7"    "TNFAIP6"  "TNFSF10"  "TOR1B"    "TREX1"    "TRIM22"  
## [49] "UBE2L6"   "XAF1"     "XIST"     "ZCCHC2"
```

As a quality control step, the size distribution of the active genesets can be visualized by using the show\_active\_genesets\_hist() function. If the input file (.gmt) is improperly formatted, you may see that all terms have the same size, or that there are fewer terms loaded than expected.

```
show_active_genesets_hist()
```

```
length(active_genesets)
```

```
## [1] 604
```

# Term Enrichment for User-Defined Terms

From the above example of adding a new resource, you can see that the geneset formats used by RITAN are simple lists. Thus, if you have your own list of interest, it can be added to RITAN and used in enrichment tests alongside other resources such as GO. For example, if you wanted to define your own term for how low-density lipoprotein (LDL) is brought into cells:

```
geneset_list$LDL = list( LDL_import = c('APOB','APOE','LDLR'), 
                         LDL_processing = c('HMGR','ACAT2','HMGCS1',
                                            'HMGCR','MVD','MVK',
                                            'PMVK','IDI1','IDI2') )
```

There are now two terms within the “LDL” resource that can be used in term enrichment by:

```
e <- term_enrichment( gs, resources = c('GO','LDL') )
```

# References

1. Ashburner M, Ball CA, Blake JA, Botstein D, Butler H, Cherry JM, Davis AP, Dolinski K, Dwight SS, Eppig JT et al: Gene ontology: tool for the unification of biology. The Gene Ontology Consortium. Nat Genet 2000, 25(1):25-29.
2. Schaefer CF, Anthony K, Krupa S, Buchoff J, Day M, Hannay T, Buetow KH: PID: the Pathway Interaction Database. Nucleic Acids Res 2009, 37(Database issue):D674-679.
3. Kanehisa M, Goto S: KEGG: kyoto encyclopedia of genes and genomes. Nucleic Acids Res 2000, 28(1):27-30.
4. Kanehisa M, Goto S, Sato Y, Furumichi M, Tanabe M: KEGG for integration and interpretation of large-scale molecular data sets. Nucleic Acids Res 2012, 40(Database issue):D109-114.
5. Croft D, O’Kelly G, Wu G, Haw R, Gillespie M, Matthews L, Caudy M, Garapati P, Gopinath G, Jassal B et al: Reactome: a database of reactions, pathways and biological processes. Nucleic Acids Res 2011, 39(Database issue):D691-697.
6. Cerami EG, Gross BE, Demir E, Rodchenkov I, Babur O, Anwar N, Schultz N, Bader GD, Sander C: Pathway Commons, a web resource for biological pathway data. Nucleic Acids Res 2011, 39(Database issue):D685-690.
7. Subramanian A, Tamayo P, Mootha VK, Mukherjee S, Ebert BL, Gillette MA, Paulovich A, Pomeroy SL, Golub TR, Lander ES et al: Gene set enrichment analysis: a knowledge-based approach for interpreting genome-wide expression profiles. Proc Natl Acad Sci U S A 2005, 102(43):15545-15550.
8. Chaussabel D, Quinn C, Shen J, Patel P, Glaser C, Baldwin N, Stichweh D, Blankenship D, Li L, Munagala I et al: A modular analysis framework for blood genomics studies: application to systemic lupus erythematosus. Immunity 2008, 29(1):150-164.
9. Li S, Rouphael N, Duraisingham S, Romero-Steiner S, Presnell S, Davis C, Schmidt DS, Johnson SE, Milton A, Rajam G et al: Molecular signatures of antibody responses derived from a systems biology study of five human vaccines. Nat Immunol 2014, 15(2):195-204.
10. Kandasamy K, Mohan SS, Raju R, Keerthikumar S, Kumar GS, Venugopal AK, Telikicherla D, Navarro JD, Mathivanan S, Pecquet C et al: NetPath: a public resource of curated signal transduction pathways. Genome Biol 2010, 11(1):R3.
