# Supplementary material for: RITAN: rapid integration of term annotation and network resources
Source: PeerJ. 2019 Jul 19;7:e6994. doi: 10.7717/peerj.6994 (PMC6644632; doi:10.7717/peerj.6994)
Supplement: Supplemental Information 1 — This archive contains five vignettes showing aspects of RITAN’s use. They are included for completeness of this manuscript as a stand-alone work. For the very latest versions, see the project’s GitHub page and/or the devel branch of bioconductor.org. [file peerj-07-6994-s001.zip › multi_tissue_analysis.html]

Multi-Tissue Analysis


# Multi-Tissue Analysis

#### *Michael T. Zimmermann*

#### *2018-10-10*

In this vignette, we will analyze a gene expression dataset with samples from multiple tissues. We will: *download a public dataset* identify the genes expressed in two tissues *run enrichment analysis, cognizant of each tissues’ expression profile* visualize network-based relationships between the tissues’ expression profiles

# Enrichment to Identify Tissue-Specific Patterns

We will use data from BgeeDB normal-tissue expression. In research, we will typically want to compare normal to one or more treatment or disease groups. Thus, consider this as an illustrative example.

```
# Load RITAN
library(RITANdata)
library(RITAN)

# Install the Bgee package. GO.db is a dependency of a dependency and may need to be installed seperately.
for (pkg in c('GO.db','BgeeDB','biomaRt')){
  if (! (pkg %in% rownames(installed.packages()) )){
    if (!requireNamespace("BiocManager", quietly=TRUE))
        install.packages("BiocManager")
    BiocManager::install(pkg)
  }
  library(pkg, character.only = TRUE)
}

for (pkg in c('tidyselect','venn','magrittr','ggplot2','igraph')){
  if (! (pkg %in% rownames(installed.packages()) )){
    install.packages(pkg)
  }
  library(pkg, character.only = TRUE)
}

# Setup Bgee query & get data (this may take some time)
bgee <- Bgee$new(species = "Homo_sapiens", dataType = "rna_seq", release = "13.2")
data <- getData(bgee)
e    <- formatData(bgee, data[[1]], callType = "present", stats = "rpkm")

# Explore the dataset with: str(sampleNames(e)), str(featureNames(e)), str(phenoData(e))
table(phenoData(e)@data$Anatomical.entity.name)

## -------------------- -
## Get expression in two tissues
tmp <- exprs(e)[ , phenoData(e)@data$Anatomical.entity.name == "heart" ]
i   <- apply( tmp, 1, function(x){ any(is.na(x)) })
expr_heart <- tmp[ !i, ]

tmp <- exprs(e)[ , phenoData(e)@data$Anatomical.entity.name == "skeletal muscle tissue" ]
i   <- apply( tmp, 1, function(x){ any(is.na(x)) })
expr_skele <- tmp[ !i, ]

venn::venn( list(Heart = rownames(expr_heart),
                 Skeletal = rownames(expr_skele) ),
            cexil= 1, cexsn = 1, zcolor = "style" )

## -------------------- -
ensembl <- useMart("ensembl", dataset = "hsapiens_gene_ensembl", "http://Aug2017.archive.ensembl.org" ) # version 90

map_heart <- getBM( attributes = c('ensembl_gene_id','ensembl_transcript_id','hgnc_symbol'),
                    filters = 'ensembl_gene_id', values = rownames(expr_heart), mart = ensembl )

map_skele <- getBM( attributes = c('ensembl_gene_id','ensembl_transcript_id','hgnc_symbol'),
                    filters = 'ensembl_gene_id', values = rownames(expr_skele), mart = ensembl )

## -------------------- -
## Functions associated with each tissue's top genes
## Important: the p-values reported here are observational, not inferential.

mh <- apply( expr_heart, 1, mean )
top_heart <- map_heart$hgnc_symbol[ map_heart$ensembl_gene_id %in% rownames( expr_heart )[ mh > quantile(mh, .975) ] ] %>% setdiff(.,'')

ms <- apply( expr_skele, 1, mean )
top_skele <- map_skele$hgnc_symbol[ map_skele$ensembl_gene_id %in% rownames( expr_skele )[ ms > quantile(ms, .975) ] ] %>% setdiff(.,'')

e <- term_enrichment_by_subset( list( Heart    = top_heart,
                                      Skeletal = top_skele ),
                                resources = 'GO_slim_PIR', all_symbols = cached_coding_genes )

plot( e[ apply(e[, c(3:4)], 1, max) >= 12, ], cap=40, label_size_y = 8, wrap_y_labels = FALSE )

## -------------------- -
## Network Interactions Within Each Tissue

net_h <- network_overlap( top_heart, resources = c('CCSB','dPPI','HumanNet') )
net_s <- network_overlap( top_skele, resources = c('CCSB','dPPI','HumanNet') )

net2g <- function(x){
  edges <- as.matrix( x[, c(1,3)] )
  G <- igraph::make_undirected_graph( c(t(edges)) )
  return(G)
}

g_h <- net2g( net_h )
g_s <- net2g( net_s )

g_dif <- igraph::difference( g_h, g_s )
g_int <- igraph::intersection( g_h, g_s )

cat(sprintf('
Of the top expressed genes, %d are shared and %d differ.
', length(V(g_int)), length(V(g_dif)) ))

par(mar=rep(0,4))
plot(g_dif, vertex.size = 2, vertex.label = NA, vertex.frame.color = 'white', layout = layout_nicely )
```
