# Supplementary material for: RITAN: rapid integration of term annotation and network resources
Source: PeerJ. 2019 Jul 19;7:e6994. doi: 10.7717/peerj.6994 (PMC6644632; doi:10.7717/peerj.6994)
Supplement: Supplemental Information 1 — This archive contains five vignettes showing aspects of RITAN’s use. They are included for completeness of this manuscript as a stand-alone work. For the very latest versions, see the project’s GitHub page and/or the devel branch of bioconductor.org. [file peerj-07-6994-s001.zip › resource_relationships.pdf]

Relationships Among Resources

Michael T. Zimmermann

2018-05-02

Identify relationships between genesets

A feature of RITAN is the ability to account for false-discovery rates across resources. However, the relationships among genesets is not always obvious. That is, two resources may each have a term that is highly similar if not identical to the other. Ideally, you would pick one of these terms to use, prior to analysis. The function geneset_overlap() helps with this process by identifying terms that have considerable overlap and merging them.

```
library(RITANdata)
library(RITAN)
```

```
# Show the fraction of genes common to multiple disease-gene relationships.
o <- geneset_overlap( geneset_list$DisGeNet )
plot( density(c( o[upper.tri(o)] )), log='y', ylim = c(1e-3, 1e3),
      main='', xlab='Fraction Shared Genes', ylab='Prob()')
```

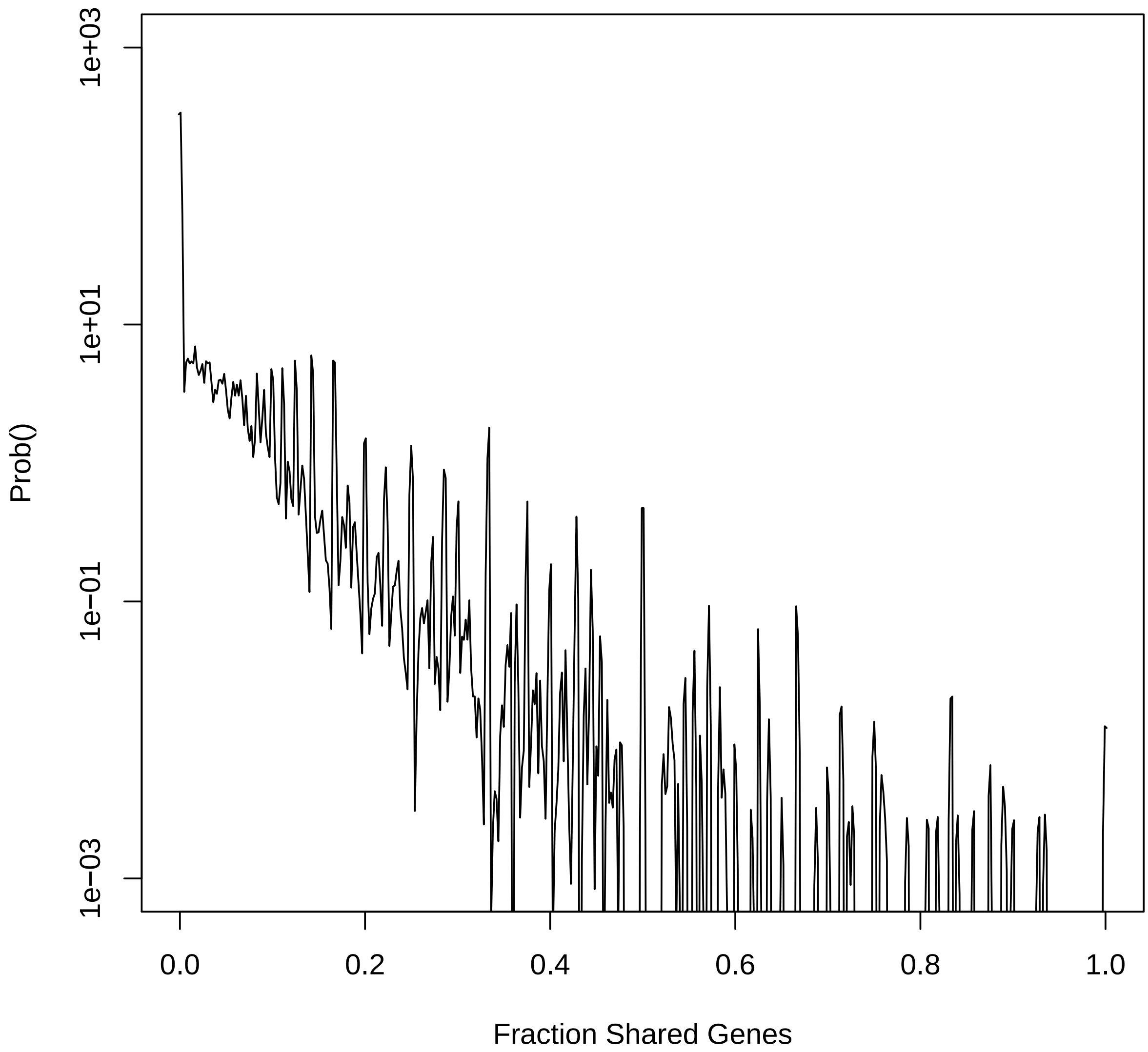


```r
# Show the diseases and their gene-level overlap, when each disease has some overlap of at least 80%.
diag(o) <- NA # ignore self-overlap
i <- which( o > 0.8, arr.ind = TRUE )
mat <- o[ unique(i[,1]), unique(i[,2]) ]
heatmap( mat, col = rev(gray(seq(0,1,length.out = 15))),
         cexRow=.7, cexCol=0.7, margins = c(7,7) )
```

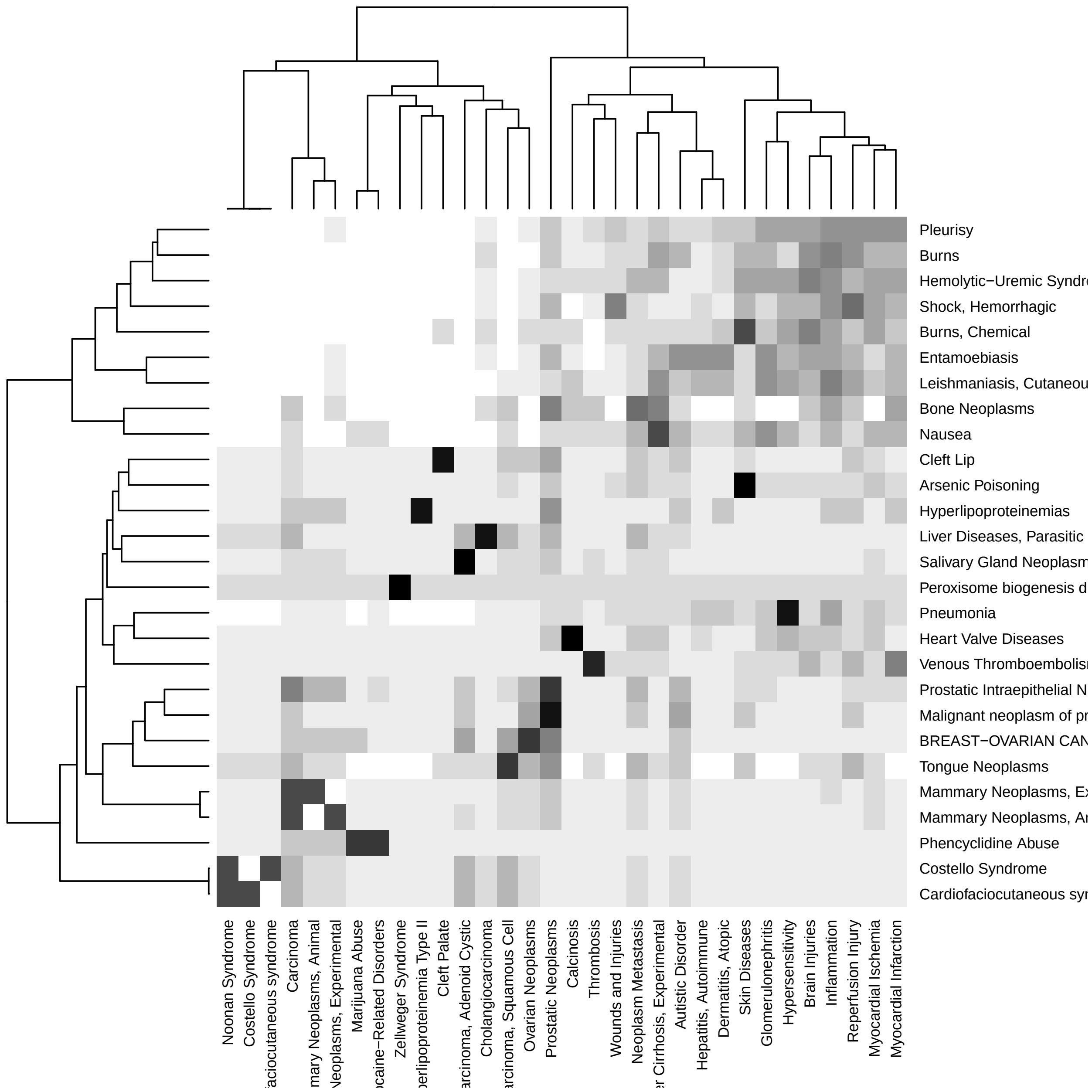


```
# Show the fraction of genes common between disease-gene relationships and GO-Slim term definitions
o <- geneset_overlap( geneset_list$DisGeNet, geneset_list$GO_slim_generic )
o <- o[ , !( colnames(o) %in% c('biological_process', 'molecular_function', 'cellular_component')) ] #
plot( density(c(o)), log='y',
      main='', xlab='Fraction Shared Genes', ylab='Prob()')
```

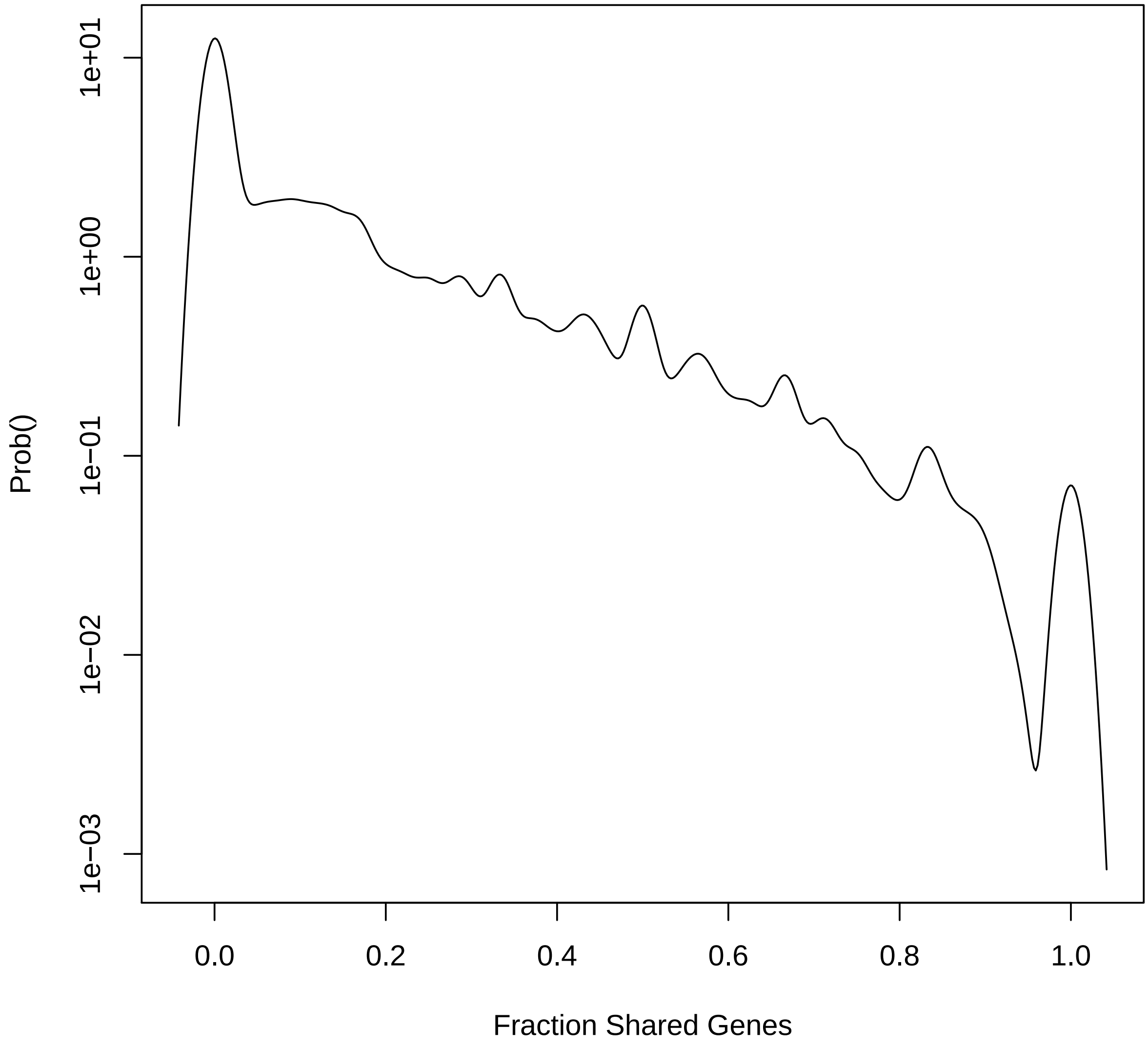


```
# Show the heatmap for relationships where a disease and term share 95% of genes in common
i <- which( o > 0.95, arr.ind = TRUE )
mat <- o[ unique(i[,1]), unique(i[,2]) ]
heatmap( mat, col = rev(gray(seq(0,1,length.out = 15))),
         cexRow=.7, cexCol=0.7, margins = c(7,7) )
```

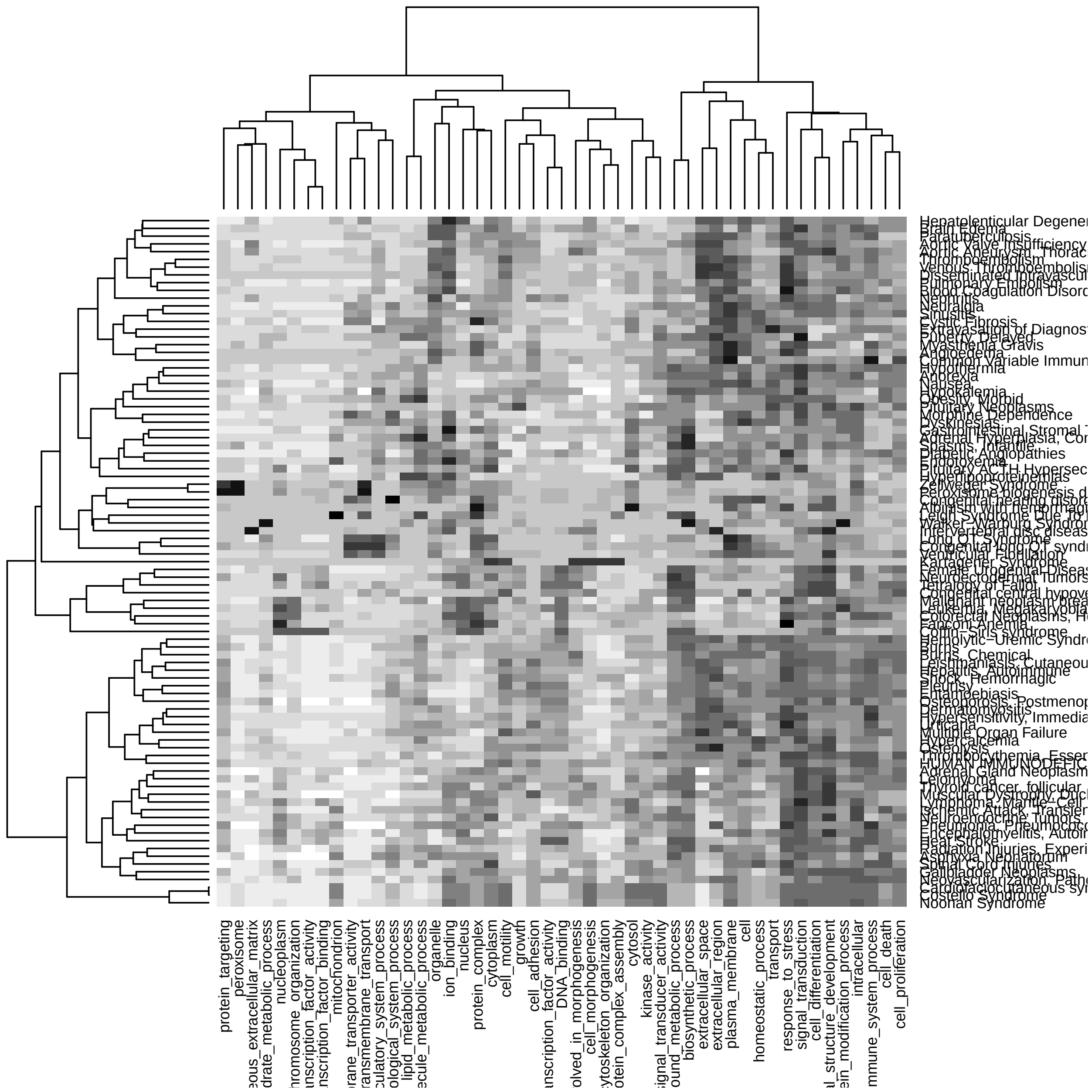

The overlapping genesets can themselves be highly informative. For instance, perhaps the diseases known to involve genes responsible for chromosome organization is of great interest to you. You can combine the available resources in RITAN to rapidly identify this intersection of genesets.

```
rownames(o)[ o[ , "chromosome_organization" ] > 0.66 ]
```

```
## [1] "Coffin-Siris syndrome"
## [2] "Leukemia, Myeloid"
## [3] "Leukemia, Megakaryoblastic, of Down Syndrome"
## [4] "Colorectal Neoplasms, Hereditary Nonpolyposis"
## [5] "Malignant neoplasm breast"
```

Thus, having many term resources in one place, such as RITAN, facilitates knowledge integration.

Features such as these can be combined to investigate the relationships among knowledge resources and the results used in further studies.

The combination of diseases and function relationships (e.g. diseases related to cell motility) can be combined to make a new geneset. This new geneset can then be used within term_enrighment() to annotate your own datasets.

```
d <- rownames(o)[ o[ , "cell_motility" ] > 0.66 ]
str(d)
```

```
##  chr [1:20] "Kartagener Syndrome" "Thromboembolism" ...
```

```
new_geneset <- intersect( unique(unlist(geneset_list$DisGeNet[d])),
                          unique(unlist(geneset_list$GO_slim_generic$cell_motility)) )
str(new_geneset)
```

```
##  chr [1:86] "CCDC40" "DNAH5" "DNAI1" "DRC1" "DYX1C1" "F2" "F7" "GAS6" ...
```

We have made a function named resource_reduce, to assist users in focusing on the unique portion of geneset resources.

```
unique_diseases <- resource_reduce( geneset_list$DisGeNet )
```

```
##
## The input list had 604 terms/genesets.
## The 55 terms with overlaps of 0.80 were merged into 22 composite terms.
## The updated term list with 571 terms will be returned.
```

```
unique_disease_slim <- resource_reduce( c(geneset_list$DisGeNet, geneset_list$GO_slim_generic), min_ove
```

```
##
## The input list had 746 terms/genesets.
## The 326 terms with overlaps of 0.95 were merged into 29 composite terms.
## The updated term list with 449 terms will be returned.
```

After running resource_reduce(), the new terms/genesets share less than min_overlap fraction of genes. If min_overlap is set to a high threshold, there will still be strong relatedness within the dataset, but redundant terms will be merged. If min_overlap is set to a lower threshold, the resulting terms will be largely independent, but it is likely that they will be a smaller number of very large terms/genesets (many mergers).

The multiple testing correction used in RITAN will be overly conservative if there are relationships between the terms used.

How stringent to be depends on your application - how the data will be annotated and interpreted.
