# Supplementary material for: RITAN: rapid integration of term annotation and network resources
Source: PeerJ. 2019 Jul 19;7:e6994. doi: 10.7717/peerj.6994 (PMC6644632; doi:10.7717/peerj.6994)
Supplement: Supplemental Information 1 — This archive contains five vignettes showing aspects of RITAN’s use. They are included for completeness of this manuscript as a stand-alone work. For the very latest versions, see the project’s GitHub page and/or the devel branch of bioconductor.org. [file peerj-07-6994-s001.zip › subnetworks.html]

Network Biology (Induced Subnetwork) Vignette


# Network Biology (Induced Subnetwork) Vignette

#### *Michael T. Zimmermann*

#### *2018-10-10*

# Introduction

RITAN is a R package intended for Rapid Integration of Term Annotation and Network resources to be applied to gene sets generated by current analysis methods.

For a general introduction to the package, please see the “enrichment” vignette.

```
library(RITANdata)
library(RITAN)
require(knitr)
```

The R object, *network\_list*, contains 6 human network-biology resources. Additionally, RITAN leverages existing R packages to access data from STRING. [In 08-2018, ProNet was depricated and internal support for HPRD and BioGRID using ProNet APIs was lost. We have added to our recommended setup script, instructions for adding both of these resources to RITAN.]. Citation information for each resource can be accessed via attr():

```
kable( attr(network_list, 'network_data_sources') )
```

| Abbreviation | Citation |
| --- | --- |
| PID | Schaefer CF, Anthony K, Krupa S, Buchoff J, Day M, et al. (2009) PID: the Pathway Interaction Database. Nucleic acids research 37: D674-679. |
| TFe | Yusuf D, Butland SL, Swanson MI, Bolotin E, Ticoll A, et al. (2012) The transcription factor encyclopedia. Genome biology 13: R24. |
| dPPI | Vinayagam A, Stelzl U, Foulle R, Plassmann S, Zenkner M, et al. (2011) A directed protein interaction network for investigating intracellular signal transduction. Science signaling 4: rs8. |
| HPRD | Prasad TS, Kandasamy K, Pandey A (2009) Human Protein Reference Database and Human Proteinpedia as discovery tools for systems biology. Methods in molecular biology 577: 67-79. |
| CCSB | Prasad TS, Kandasamy K, Pandey A (2009) Human Protein Reference Database and Human Proteinpedia as discovery tools for systems biology. Methods in molecular biology 577: 67-79. |
| STRING | Szklarczyk D, Franceschini A, Kuhn M, Simonovic M, Roth A, et al. (2011) The STRING database in 2011: functional interaction networks of proteins, globally integrated and scored. Nucleic acids research 39: D561-568. |
| HumanNet | Lee I, Blom UM, Wang PI, Shim JE, Marcotte EM. Prioritizing candidate disease genes by network-based boosting of genome-wide association data. Genome Res. 2011;21(7):1109-1121. |
| Biogrid | Chatr-Aryamontri A, Breitkreutz BJ, Oughtred R, Boucher L, Heinicke S, Chen D, Stark C, Breitkreutz A, Kolas N, ODonnell L, Reguly T, Nixon J, Ramage L, Winter A, Sellam A, Chang C, Hirschman J, Theesfeld C, Rust J, Livstone MS, Dolinski K, Tyers M. The BioGRID interaction database: 2015 update. Nucleic Acids Research. Nov. 2014 |
| ChEA | Lachmann A, Xu H, Krishnan J, Berger SI, Mazloom AR, Maayan A. (2010) ChEA: transcription factor regulation inferred from integrating genome-wide ChIP-X experiments. Bioinformatics. Oct 1;26(19):2438-44. |

\*NOTE that many of these resources are themselves combinations of other more specialized resources.

### We provide simple access to multiple network biology resources in order to facilitate rapid evaluation, basic data annotation, and integration using a common framework.

### We will elaborate on this functionality in the following examples.

---

# Example 1 - Basic Network Query

We begin by gathering a starting list of gene symbols. In this example, we choose the genes that are most highly up-regulated in human PBMCs, compared to healthy controls, gathered from an MSigDB module. See our “enrichment” vignette for further introduction to MSigDB. This example, named *my\_genes* below, is intended to represent the outcome of an analysis - the genes identified by a high-throughput experiment. RITAN facilitates the integration of multiple network biology resources to query the know interactions within *my\_genes*.

```
my_genes <- geneset_list$MSigDB_C7[['GSE6269_HEALTHY_VS_FLU_INF_PBMC_DN']]
net <- network_overlap( my_genes, resources = 'CCSB' )
```

```
## 
## Generating undirected subnetwork...
## 
## Removing duplicate edges and self-loops...
## Total induced subnetwork from 160 genes has 7 nodes and 4 edges (4 unique).
```

We now have what is referred to as the “induced subnetwork” for *my\_genes* using only one resoure, CCSB. The network is the subset of interactions from CCSB that contains exclusively gene symbols within *my\_genes*.

The input list contained 160 symbols and 7 were identified as having interactions within the network resources. Note: If very few genes have known connections between them, there may be a mismatch between your input format and the network resources - see the “Check Input” section below.

There are 4 edges returned by this query with the first 5 shown for example:

```
head(unique(net))
```

```
##           p1 edge_type    p2
## 4911   GNAI2      CCSB GPSM3
## 5836   IFIT1      CCSB IFIT3
## 6027   ISG15      CCSB USP18
## 10118 PLSCR1      CCSB GNAI2
```

The output format is a data.frame with three columns:

| Name | Description |
| --- | --- |
| p1 | The first interacting protein |
| edge\_type | The manner in which p1 and p2 interact |
| p2 | The second interacting protein |

The edge type is taken from the network resource, when possible. For instance, the first edge shown above indicates the self-reaction of the gene WAS. The second edge indicates protein-protein interaction between WAS and GRB2.

---

# Example 2 - Filtering out low-confidence interactions

Some network resources provide confidence scores for each interaction. Most notably, the popular STRING resource has indexed many other resources and additionally included computationally inferred interactions. Thus, many of the interactions within STRING have not been experimentally validated. While that does not mean they are incorrect, depending on your application you may not be comfortable with including them. We provide a simple means for trimming resources that provide scores using a simple threshold.

```
net2 <- network_overlap( my_genes, resources = c('CCSB','STRING'), minStringScore = 700 )
str(net2)
```

Adding STRING brings the network to 86 genes and 265 edges among them. Thus, considering additional resources increases the coverage of the input set of genes.

Scores within STRING are on a [0, 1000] scale with 1000 taken as “fully confident.” We recommend using a threshold of 700 (the default) for “high confidence” investigation. The further integration of multiple resources (as performed here) will help to achieve breadth of coverage without resorting to inclusion of low-confidence interactions.

RITAN currently indexes 9 resources which can be quiried individually, or together to achieve greater coverage. As each resource is derived from different types of experiments, consider if the source and interaction type are applicable to your dataset. For a reference listing for resources, see attr(network\_list, ‘network\_data\_sources’).

---

# Check Input

### Check how many input gene symbols are within the network resources.

A good quality-control step is to check how many of your input genes of interest actually appear in any of the network resources. If too few appear, you either have identified a set of poorly understood genes (novel finding), or the symbols used do not match (gene symbol version mismatch). The second is more likely.

To quickly check if your symbols appear in any of the loaded network resources:

```
my_genes <- geneset_list$MSigDB_C2[['VERNOCHET_ADIPOGENESIS']]
i <- check_any_net_input( my_genes )
table(i)
```

```
## i
## FALSE  TRUE 
##     1    18
```

From the above, you can see that all (save one) genes in the adipogenesis geneset appear in a network resource. Thus, we can be confident that that the combined network resource well-represents our genes of interest. Additionally, the gene symbols in use by our input study (in this case, MSigDB) and RITAN, are compatible.

Users may want to only use one or a few selected resources. This may be useful for many reasons including comparison to previous literature or preference for direct physical interactions over regulatory interactions. To perform the analogous check using one resource:

```
i <- check_net_input( my_genes, network_list[['dPPI']] )
table(i)
```

```
## i
##  no yes 
##   9  10
```

```
names(i)[i == 'no']
```

```
## [1] "AOC3"    "APCDD1"  "CYP2F1"  "LUM"     "PANK3"   "RARRES2" "RETN"   
## [8] "SULT1A1" "VNN3"
```

Now, it is evident that while across the resources indexed by RITAN, all of the genes are represented, the representation by any one resource may be less comprehensive. This is the primary reason that we have provided multiple resources within RITAN - to achieve more comprehensive coverage.

---

# Example 3 - Including Neighbors

In many instances, the known relationships within a group of genes is the most direct (as above). However, a broader analysis considering what other genes interact with the input list may be informative. If only a few top candidates are of interest, prioritized by computational methods, we suggest established tools such as GeneMANIA (webserver or Cytoscape App) or CyTargetLinker. RITAN will provide all neighbors, with stringency filtering provided.

Because the previous example has a large number of genes, we will switch to a smaller example. Again leveraging MSigDB, we gather an example of 19 genes “up-regulated with adipogenic differentiation and down-regulated by troglitazone.”

```
my_genes <- geneset_list$MSigDB_C7[['GOLDRATH_NAIVE_VS_MEMORY_CD8_TCELL_UP']]
net3.1 <- network_overlap( my_genes, resources = 'PID',
                           include_neighbors = FALSE, dedup = TRUE )
```

```
## 
## Generating undirected subnetwork...
## 
## Removing duplicate edges and self-loops...
## Total induced subnetwork from 200 genes has 6 nodes and 4 edges (4 unique).
```

```
nets2use <- c('PID','dPPI','TFe','HumanNet','CCSB')
net3.2 <- network_overlap( my_genes, resources = nets2use,
                           include_neighbors = FALSE, dedup = TRUE )
```

```
## 
## Generating undirected subnetwork...
## 
## Removing duplicate edges and self-loops...
## Total induced subnetwork from 200 genes has 128 nodes and 241 edges (241 unique).
```

```
net3.3 <- network_overlap( my_genes, resources = 'PID',
                           include_neighbors = TRUE, dedup = TRUE )
```

```
## 
## Generating undirected subnetwork...
## 
## Removing duplicate edges and self-loops...
## Total induced subnetwork from 200 genes has 420 nodes and 2608 edges (2608 unique).
```

First, we identified the induced subnetwork about our geneset of interest within the Pathway Interaction Database (PID). We added the *dedup* option to remove duplicate edges.

Second, we augmented this subnetwork by using multiple network resources. As in the previous examples, the coverage of the input geneset is markedly increased.

Third, we augmented the PID-only network by including all of the first-neighbors of input genes. The “small-world” and context-non-specific nature of these network resources can easily lead to massive networks with thousands of genes included, making interpretation very difficult (a.k.a. unwieldy “hairballs”). Thus, we recommend pre-planned and thought-out analysis plans, when possible, to focus your network-based study on the most relevant interactions.

The neighbors included in lists such as net3.3 indicate candidates for further study that could modulate the activity of, in this case, differentially expressed genes.

---

# Example 4 - Visualize Network within R

RITAN facilitates generating or gathering a network, while existing methods exist for analysis of the network. One solution is the igraph package which provides a wide range of network analysis and visualization functionality. For brevity, we will not query the larger network resources here.

From the following example plot, we can identify HIF1-alpha as a hub gene within the Memory-CD8-T-cell up-regulated genes loaded in the previous example.

```
require(igraph)
```

```
## Loading required package: igraph
```

```
## 
## Attaching package: 'igraph'
```

```
## The following objects are masked from 'package:stats':
## 
##     decompose, spectrum
```

```
## The following object is masked from 'package:base':
## 
##     union
```

```
net4 <- network_overlap( my_genes, resources = c('PID','dPPI','TFe'),
                         include_neighbors = FALSE, dedup = TRUE )
```

```
## 
## Generating undirected subnetwork...
## 
## Removing duplicate edges and self-loops...
## Total induced subnetwork from 200 genes has 31 nodes and 24 edges (24 unique).
```

```
edges <- as.matrix( net4[, c(1,3)] )
G <- igraph::make_undirected_graph( c(t(edges)) )
par(mar=rep(0,4))
plot(G, vertex.size = 20, vertex.frame.color = 'white' )
```

---

# Example 5 - Identify “Bridging” Genes/Proteins

RITAN is a generic tool that can be used with other tools to facilitate novel analyses.

Say you have two genes of interest that do not interact with one another, but you suspect that they influence one another. You can use the igraph package with RITAN to investigate this question.

Below, we show how to identify “bridging” genes/proteins from a network using the example of two cell cycle checkpoint proteins, TP53 and SFN.

```
require(igraph)

G <- as.graph( network_list$PID )
all( c("EP300", "SFN", "TP53", "CCNB1") %in% names(V(G)) )
```

```
## [1] TRUE
```

```
get.shortest.paths(G, "TP53" , to="CCNB1", mode = "all" )$vpath
```

```
## [[1]]
## + 2/1980 vertices, named, from f0c196d:
## [1] TP53  CCNB1
```

```
get.shortest.paths(G, "EP300", to="SFN"  , mode = "all" )$vpath
```

```
## [[1]]
## + 4/1980 vertices, named, from f0c196d:
## [1] EP300    MAX      HSP90AA1 SFN
```

```
G <- as.graph( network_list$HumanNet )
all( c("EP300", "SFN", "TP53", "CCNB1") %in% names(V(G)) )
```

```
## [1] TRUE
```

```
get.shortest.paths(G, "TP53" , to="CCNB1", mode = "all" )$vpath
```

```
## [[1]]
## + 3/16155 vertices, named, from f0f775d:
## [1] TP53  CCNG1 CCNB1
```

```
get.shortest.paths(G, "EP300", to="SFN"  , mode = "all" )$vpath
```

```
## [[1]]
## + 3/16155 vertices, named, from f0f775d:
## [1] EP300 ABL1  SFN
```

---

# Example 6 - Write data for import into Cytoscape

Cytoscape is a powerful platform for network analysis that has gathered a large following from divers fields of application. Thus, many extensions (Apps) have been written for Cytoscape. RITAN facilitates the generation of networks using integrated resources and those networks can be exported for use in Cytoscape. Beginning from one of the induced subnetworks generated above, we demonstrate export of this data to simple text files and how it can be imported into Cytoscape for visualization and presentation.

The publication for the geneset is available here and describes which genes are “induced by white adipocyte differentiation and inhibited by troglitazone activation of PPAR-gamma.” Taking data from Table 1, we annotate the genes by functional data. Additionally, we add a variable to indicate which genes are within the initial list of genes.

```
my_genes <- geneset_list$MSigDB_C2[['VERNOCHET_ADIPOGENESIS']]
net5 <- network_overlap( my_genes )
 g <- unique(c( net5$p1, net5$p2 ))

tab <- data.frame( gene      = c('FABP4',  'CEBPA','PPARG','ADRB3','RETN','AGT','HP',
                                 'RARRES2','PANK3','FFAR2','LUM',  'MC2R','ADCYAP1R1'), 
                   TrogRatio = c( 1.8,      1.7,    0.6,    0.3 ,   0.3 ,  0.4  ,0.2, 
                                  0.3,      0.1,    0.5,    0.3 ,   0.5 ,  0.1),
                   WAT_BAT   = c( 0.8,      1.0,    0.6,    10.0,   21.6,  215.4,2.4, 
                                  9.5,      3.9,    4.6,    4.0 ,   7.3 ,  2.6),
                   initial   = g %in% my_genes
                   )

write_simple_table(net3.1, 'net_example.sif') 
write_simple_table(tab,    'net_example.tab')
```

The `write_simple_table` will produce plain text files for input into Cytoscape.

**Briefly**, these can be imported into Cytoscape by:

- Import the network with: File –> Import –> Network –> File…
- Import the data table with: File –> Import –> Table –> File…

**See the Cytoscape Tutorial** for thorough examples of importing data into Cytoscape.

---

# Example 7 - Use Your Own Network Resource

For many reasons, you may wish to use a network resource that we have not provided with this package. Some examples include use of a different ID system, non-human data, or use of a gene coexpression network. In this example, we use a short example of genes with tissue-specific expression from Tang’s 2010 Nature paper, and the mouse interaction network from BioGRID.

```
## Add a new resource to "network_list"
### For brevity, we
network_list[['BioGRID_Mouse']] <- readSIF( 'BIOGRID-ORGANISM-Mus_musculus-3.4.136.symbols.sif.gz', header = TRUE )
# > str(network_list[['BioGRID_Mouse']])
# 'data.frame': 38322 obs. of  3 variables:
#  $ p1       : chr  "SMAD2" "SMAD2" "SMAD2" "SMAD2" ...
#  $ edge_type: chr  "physical" "physical" "physical" "physical" ...
#  $ p2       : chr  "Rasd2" "Rab34" "Rhebl1" "Rab38" ...

## Short example from Tang's 2010 Nature paper
my_mouse <- c('Sost','Fxyd4','Tmprss6','Crtap','Thpo','Kcnn4','Osm','Slc29a3','ALB')

## First, check if these genes appear in the BioGRID network.
check_net_input( my_mouse, network_list[['BioGRID_Mouse']] )
#  Sost   Fxyd4 Tmprss6   Crtap    Thpo   Kcnn4     Osm Slc29a3     ALB
# "yes"    "no"    "no"    "no"    "no"    "no"    "no"    "no"    "no"

## After correcting a few gene names, get the induced subnetwork from mouse data.
my_mouse <- c('Sost','Fxyd4','Tmprss6','CRTAP','Thpo','KCNN4','Osm','Slc29a3','ALB')
net.m <- network_overlap( my_mouse, include_neighbors = TRUE, resources = c('BioGRID_Mouse') )
str(net.m)
# Generating undirected subnetwork...
# Total induced subnetwork from 9 genes has 17 nodes and 17 edges (17 unique).
# 'data.frame': 17 obs. of  3 variables:
#  $ p1       : chr  "Sf3a1" "Nphp1" "Iqcb1" "Invs" ...
#  $ edge_type: chr  "physical" "physical" "physical" "physical" ...
#  $ p2       : chr  "CRTAP" "Invs" "Nphp1" "ALB" ...

## Also, check within BioGRD's human network
check_net_input( my_mouse, network_list[['BioGRID_Human']] )
# Sost   Fxyd4 Tmprss6   CRTAP    Thpo   KCNN4     Osm Slc29a3     ALB
# "no"    "no"    "no"   "yes"    "no"   "yes"    "no"    "no"   "yes"

## Note that gene symbols are case sensitive
my_mouse <- c('SOST','Fxyd4','Tmprss6','CRTAP','THPO','KCNN4','OSM','Slc29a3','ALB')
check_net_input( my_mouse, network_list[['BioGRID_Human']] )
#  SOST   Fxyd4 Tmprss6   CRTAP    THPO   KCNN4     OSM Slc29a3     ALB 
# "yes"    "no"    "no"   "yes"    "no"   "yes"   "yes"    "no"   "yes"

## Get the induced subnetowrk from human data
net.h <- network_overlap( my_mouse, include_neighbors = TRUE, resources = c('BioGRID_Human') )
str(net.h)
# Generating undirected subnetwork...
# Total induced subnetwork from 9 genes has 224 nodes and 755 edges (634 unique).
# 'data.frame': 755 obs. of  3 variables:
#  $ p1       : chr  "MBIP" "SH3GL1" "TNNT1" "GFAP" ...
#  $ edge_type: chr  "physical" "physical" "physical" "physical" ...
#  $ p2       : chr  "MBIP" "SH3GL1" "TNNT1" "GRAP2" ...
```

From comparison of the above results, there are noticeable differences between using each resource. Some genes are present in the mouse interaction network that are not present in the human (e.g. Fxyd4). Also, there is usually a greater extent of interaction data available for human genes/proteins. This may be more helpful for some studies, but too indirect for others.

---

# Example 8 - Putting it All Together

**RITAN’s two primary functions can be used together in order to gain stronger insights into the functional role of the identified gene(s).**

Between the two RITAN vignettes, you’ve seen how to use RITAN to perform term enrichment and to generate subnetworks leveraging multiple data sources. In the following example, we will start from a single gene of interest, gather the genes that it is known to physically interact with (protein-protein interaction; PPI), and then perform enrichment analysis on group of interacting genes/proteins.

```
net <- network_overlap( 'FOXP3', include_neighbors = TRUE, resources = c("PID","dPPI","CCSB" ) )
```

```
## 
## Generating undirected subnetwork...
## 
## Removing duplicate edges and self-loops...
## Total induced subnetwork from 1 genes has 10 nodes and 21 edges (21 unique).
```

```
genes <- unique(c( net$p1, net$p2 ))
e1 <- term_enrichment( genes, "Blood_Translaiton_Modules", verbose=FALSE, all_symbols = cached_coding_genes )
summary(e1)
```

```
##                                                                                       name
## 77                              Blood_Translaiton_Modules.signaling in T cells (I) (M35.0)
## 24 Blood_Translaiton_Modules.regulation of antigen presentation and immune response (M5.0)
## 50                       Blood_Translaiton_Modules.AP-1 transcription factor network (M20)
## 1                                        Blood_Translaiton_Modules.targets of FOSL1/2 (M0)
## 2                  Blood_Translaiton_Modules.integrin cell surface interactions (I) (M1.0)
## 3                 Blood_Translaiton_Modules.integrin cell surface interactions (II) (M1.1)
##               p n n.set            q
## 77 3.786623e-08 3    14 1.310171e-05
## 24 8.080113e-06 3    79 1.397860e-03
## 50 2.251947e-05 2    14 2.597246e-03
## 1  1.000000e+00 0    11 1.000000e+00
## 2  1.000000e+00 0    29 1.000000e+00
## 3  1.000000e+00 0    12 1.000000e+00
```

```
e2 <- term_enrichment( genes, "ReactomePathways", verbose=FALSE, all_symbols = cached_coding_genes )
summary(e2)
```

```
##                                                                        name
## 520                  ReactomePathways.Fc epsilon receptor (FCERI) signaling
## 489                         ReactomePathways.FCERI mediated MAPK activation
## 86  ReactomePathways.Activation of the AP-1 family of transcription factors
## 732                                   ReactomePathways.Innate Immune System
## 706                                          ReactomePathways.Immune System
## 816   ReactomePathways.MAPK targets/ Nuclear events mediated by MAP kinases
##                p n n.set            q
## 520 4.129647e-07 5   340 0.0007041047
## 489 5.373589e-06 4   246 0.0045809850
## 86  1.114849e-05 2    10 0.0063360586
## 732 1.801576e-05 5   734 0.0066978019
## 706 1.964165e-05 6  1339 0.0066978019
## 816 1.071665e-04 2    30 0.0304531344
```

Thus, starting from only one gene of interest, we identified 9 additional physically interacting genes. These genes are known to be co-regulated in a Blood-Translation-Module associated with T-cell signaling. Additionally, they are significantly associated with four Reactome pathways including AP-1 and MAPK signaling
